# Commentary: Decoding across sensory modalities reveals common supramodal signatures of conscious perception

**DOI:** 10.3389/fnhum.2020.00195

**Published:** 2020-06-11

**Authors:** Talis Bachmann

**Affiliations:** Laboratory of Cognitive Neuroscience, School of Law, University of Tartu, Tallinn, Estonia

**Keywords:** neural correlates of consciousness (NCC), consciousness states, contents of consciousness, perception, intermodal perception, cortex, thalamus

Research on neural signatures of consciousness can be divided between studies of the state of consciousness (Barttfeld et al., 2015; Demertzi et al., 2019) and contents of consciousness (Schurger et al., 2015; Webb et al., 2016), with relatively litte overlap between these two traditions (however, see Aru et al., 2019). Hardly anyone would question that state of consciousness is mediated by panmodal or intermodal mechanisms. On the other hand, signatures of the contents of consciousness have been examined typically by unimodal experimental setups, leaving open the question of whether the mechanisms of contents of consciousness are universal (i.e., panmodal/intermodal) or specific to each sensory modality such as visual, auditory, somatosensory, etc. An important step forward was taken by Sanchez et al. (2020), who revealed supramodal patterns of magnetoencephalography (MEG) signatures of contentful conscious perception, with high measures of decoding success. How did they do this?

In each modality, in the detection paradigm, individually calibrated near-threshold stimuli were presented for 50 ms. Participants had to report the presence or absence of the stimulus (with sham- and high-intensity catch trials used in addition to the stimulus presentation trials to control for various artifacts). Brain activity recorded in trials where stimulus was detected was compared with activity from trials where the stimulus was not detected. (Multivariate pattern analysis of MEG signals combined with a searchlight method was used for optimal decodability. Differences in source-level event-related fields between conscious vs. not conscious trials provided the signatures of conscious experience). Decoding analysis in the poststimulus epoch between sensory modalities revealed spatiotemporal activity patterns predicting conscious experience of the stimulus. These activity patterns, extracted also in the no-report experimental conditions, featured activity in primary sensory regions not directly relevant to the task; common signatures of conscious access to contents across sensory modalities were thereby demonstrated. Importantly, intermodal correlates of conscious access emerged relatively late poststimulus (e.g., >200 ms).

The authors (Sanchez et al., 2020) interpret their results in terms of multisensory integration and top-down broadcasting of neural representations. In other words, they seem to assume that specific informational content of different modalities interacts in a way that produces intermodal signatures of conscious perception. However, the method used by Sanchez et al. (2020) does not allow to know the *sources* of these generalized signatures of conscious access *unfolding* after 200 ms. The top-down broadcast may be either strictly cortical (perhaps mediated by intermodal synchronization; e.g., Friese et al., 2016), originating from frontal systems or thalamo-cortical (or cortico-thalamo-cortical), with afferents from the so-called non-specific thalamic and/or brain stem neurons and propagating in a phasic mode all over the cortex (especially targeting the layer-1 dendrites of the infragranular pyramidal neurons) (Bachmann and Hudetz, 2014; Phillips et al., 2018; Aru et al., 2019). In this case there is no broadcast of the *contents* to be intermodally integrated, but just a nonspecific, temporally delayed modulation universally directed at the visual, auditory, and somatosensory cortical areas.

Presumably, future research will be able to specify whether the late modally generalized activity as a signature of conscious contentful perception would be strictly cortically generated or originates from subcortical neural units not specified for transmission of sensory contents as the classical relay nuclei are (e.g., lateral geniculate). Of course, as MEG sensitivity to deep subcortical neural sources is quite less than that for the cortical units, some animal research with subcortically implanted electrodes or optogenetic devices would be commendable (e.g., Suzuki and Larkum, 2020). Whether the source of widespread modulation might be in the locus coeruleus for launching the noradrenergic boost (Phillips et al., 2018) or some other modulators might be involved (Bachmann and Hudetz, 2014) remains an open question. In one way or another, the approaches arguing and showing that besides the states of consciousness also contents of consciousness have their panmodal neural signatures (Bachmann, 2011; Sanchez et al., 2020) must be taken seriously, as they may change our general views on how the brain “makes” consciousness.

## Author Contributions

TB designed the research and wrote the paper.

## Conflict of Interest

The author declares that the research was conducted in the absence of any commercial or financial relationships that could be construed as a potential conflict of interest.
